# Long-term statin adherence in patients after hospital discharge for new onset of atherosclerotic cardiovascular disease: a population-based study of real world prescriptions in Taiwan

**DOI:** 10.1186/s12872-019-1032-4

**Published:** 2019-03-15

**Authors:** Shu-ting Chen, Shih-ting Huang, Wen-Yi Shau, Chao-Lun Lai, Jim Z. Li, Selwyn Fung, Vicki C. Tse, Mei-Shu Lai

**Affiliations:** 1 Institute of Epidemiology and Preventive Medicine, College of Public Health, National Taiwan University, No. 17, Xu-Zhou Road, Zhongzheng District, Taipei, Taiwan 10055; 2 Pfizer Inc, No 177, Zhongzheng East Road, Tamsui District, New Taipei City, Taiwan 25159; 3 Department of Internal Medicine and Center for Critical Care Medicine, National Taiwan University Hospital Hsin-Chu Branch, No. 25, Lane 442, Section 1, Jingguo Road, Hsin-Chu City, Taiwan 30059; 4 Department of Internal Medicine, College of Medicine, National Taiwan University, No. 1, Section 1, Jen-ai Road, Zhongzheng District, Taipei, Taiwan 10051; 5 Pfizer Inc, 10555 Science Center Dr, San Diego, CA 92121 USA; 6 Pfizer Inc, 235 E 42nd St, New York, NY 10017 USA; 7 APCER Life Sciences, Inc, 3 Independence Way, Suite 300, Princeton, NJ 08540 USA

**Keywords:** Atherosclerosis, Adherence, Persistence, Secondary prevention, Hypercholesterolemia, Statins, Taiwan

## Abstract

**Background:**

Despite the recommendations of statins treatment for secondary prevention of atherosclerotic cardiovascular disease (ASCVD), treatment adherence and persistence are still a concern. This study examined the real world practice of long-term adherence and persistence to statins treatment initiated after hospital discharge for ASCVD, and their associated factors in a nationwide cohort.

**Methods:**

Post discharge statin prescriptions between 2006 and 2012 were extracted from the Taiwan National Health Insurance claims database. Good adherence, defined as proportion of days covered (PDC) ≥0.8 and mean medication possession ratio (MPR), was measured every 180-day period. Non-persistence was defined on the date patients failed to refill statin for 90 days after the end of the last prescription. Their associations with influential factors were analyzed using a generalized estimating equation and Cox’s proportional hazard model.

**Results:**

There was a total of 185,252 post-discharge statin initiations (from 169,624 patients) and followed for 467,398 patient-years in the study cohort. Percentage of good adherence (mean MPR) was 71% (0.87) at 6-months; declined to 54% (0.68), 47% (0.59), and 42% (0.50) at end of year 1, 2, and 7, respectively. Persistence in statin treatment was 86, 67, 50, and 25% at 6-month, 1-, 2-, and 7-year, respectively. Comparing the statin-cohort initiated from year 2006 to 2012, 1-year persistence increased from 58 to 73%, and 1-year good adherence improved from 45 to 61%. Factors associated with sub-optimal adherence and non-persistence included: prescription by primary care clinics or non-cardiology specialties; patients’ age > 75 years; no history of previous statin use; ASCVD events with ischemic stroke diagnosis; comorbidities of renal disease, liver disease, depression, and chronic obstructive pulmonary disease.

**Conclusions:**

Despite the improving trends, long-term adherence and persistence of statin treatment were suboptimal in Taiwan. Strategies to maintain statin treatment adherence and persistence need to be implemented to further enhance the positive trend.

## Background

Non-adherence to medication is a major problem in patients with chronic conditions including cardiovascular diseases [1, 2]. Approximately 50% of patients with chronic diseases are not adherent to medications in developed countries including the United States [3]. Population-based observational studies from the United States [2, 4], Canada [1], and Taiwan [5] have shown suboptimal adherence to statin therapy. Studies conducted in the United States [6] and Germany [7] showed that persistence in statin therapy decreases over time after the therapy initiation. It was estimated that medication non-adherence lead to approximately 10% of hospital admissions, caused 125,000 deaths from cardiovascular diseases per year in the United States, and results in a cost of approximately US$100 billion per year [3, 8]. Maintaining good levels of treatment adherence and persistence for chronic conditions, which requires long-term management, is essential to maintaining the treatment effect. Information regarding the level of treatment adherence and persistence as well as their potential influential factors is important to enhance effectiveness of health care delivery.

The efficacy of statin on secondary prevention of ASCVD has been established and recommended by most recent international treatment guidelines [9–11]. Statin treatment for high-risk cardiovascular disease patients has been reimbursed by the Taiwan National Health Insurance (NHI) program since its launch in 1995. Despite the comprehensive medical service coverage in the unified NHI system [12], the level of adherence and persistence of statin treatment in the long-term is not clear. The primary aim of this study was to analyze long-term adherence and persistence of statin use in Taiwanese patients after being discharged from hospitalization for a new-onset of ASCVD. The secondary aim was to explore factors associated with statin adherence and persistence.

## Methods

### Study design

**Fig. 1:**
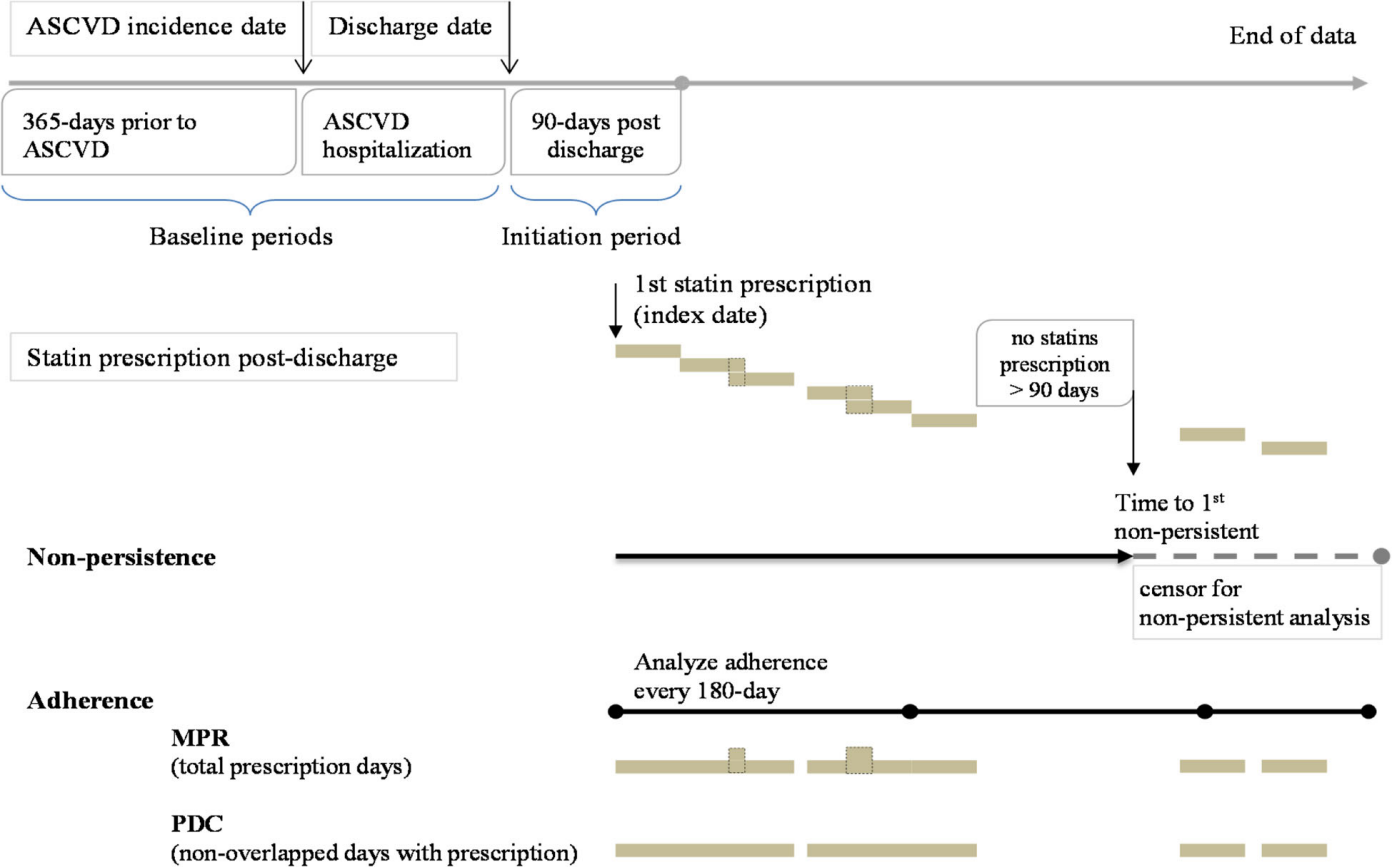
Study timeline, non-persistence, and adherence measurement

This was a population-based retrospective cohort study of patients who initiated statin within 90 days after discharged from hospitalization for a new episode of ASCVD between 2006 and 2012 using the NHI claim database. The study timeline was illustrated in Fig. 1.

### Data source and data extraction

As a compulsory universal single-payer program, the Taiwan NHI covered more than 99% of the total 23 million population as of the year 2014 [12]. The medical benefits covered by NHI include: ambulatory care, emergency room visits, hospitalization, surgical procedures, laboratory tests, diagnostic examinations, dental services, traditional Chinese medicines, and prescription medications. The reimbursement data submitted by healthcare institutes are captured electronically on a daily basis. Given the universal coverage and comprehensive medical services, the NHI claims database provides a good means for population-based medical resource utilization research and has been widely used in clinical and epidemiological studies.

Our study population was the patients aged from 20 to 100, who were discharged from hospital for a new ASCVD episode, and initiated a statin within 90 days after hospital discharge. All claims for prescriptions and medical procedures were screened to identify eligible patients from the total 23 million population covered by NHI in Taiwan. These patients’ claim data, recorded in NHI drug codes and NHI procedure codes, were extracted together with the patients’ demographics, diagnoses and their health care provider information. The ASCVD episodes were identified according to revascularization procedures performed to the patients in hospital and their discharge diagnoses. We ascertained the revascularization procedures using the NHI procedure codes for percutaneous transluminal coronary angioplasty (PTCA) and coronary artery bypass graft (CABG) in patients’ hospitalization claims. Discharge diagnoses in the database were recorded in the International Classification of Diseases, 9th Revision, Clinical Modification (ICD-9-CM) codes. We selected patients whose discharge diagnoses including one of the following three: acute myocardial infarction (AMI) (ICD-9-CM codes 410.xx), coronary heart disease (CHD) (ICD-9-CM codes 414.01), and ischemic stroke (ICD-9-CM codes 433.xx and 434.xx). To clarify the hospitalization was for a new onset, we examined all claim records of the patient up to 1 year preceding the admission to confirm no prior ASCVD episode. The validity of AMI, ischemic stroke diagnosis codes and revascularization procedures codes in the NHI claim database has been studied against clinical records in hospital setting. The results showed high positive predictive value of these reimbursement codes, which were consistent with clinical data [13, 14]. To protect confidentiality, patients’ identity data were encrypted and analyzed anonymously; all results were reported in aggregate manner and no individual data were revealed. The research ethics committee of the National Taiwan University Hospital reviewed and decided the study protocol qualified for waiving of ethics approval in compliance with governmental laws and regulations (NTUH-REC N0. 201,409,059 W). We applied for the data in year 2015, and completed the analyses in year 2017.

### Study variables

We measured statin adherence using proportion of days covered (PDC) and medication possession ratio (MPR) [15, 16]. MPR and PDC were indirect measurements of medication use derived from prescription claims. However, as all statins were reimbursed in the same universal program (NHI) during study period in Taiwan, and all prescription records were captured systematically and consistently in daily clinical practice, prescription claims were the most available data for a population based study. In contrast, direct measurements of medication use, such as level of statin or its metabolites in blood or urine, were not collected in routine clinical practice; neither was the level of biological markers in blood, such as serum lipid level, reported in the reimbursement claims. To calculate PDC and MPR, we divided patients’ follow-up durations – from statin initiation to end of data collection or another ASCVD event – into 180-day periods. We calculated MPR as the sum of statin prescription days in each 180-day period divided by number of calendar days in the period. MPR was analyzed as a continuous variable. After removing overlapped prescription days, which could happen when patient refilled a prescription before the end of the previous prescription, PDC was calculated as the proportion of non-overlapped prescription-covered days in the 180-day period; PDC was analyzed as a binary variable with ≥0.8 labelled as good adherence and < 0.8 as suboptimal adherence [17].

Statin treatment persistence was analyzed as a time-to-event variable. We defined the first non-persistence event on the date when a patient first failed to refill statin medication for 90 days after end of last prescription. The persistence time started from the index date, which was the date the first statin was prescribed within 90 days of hospital discharge. The duration between index date and the first non-persistence date was defined as statin persistence. Along follow up, if a non-persistence has not ever reached until ended of data or when another ASCVD hospitalization occurred first, the persistence data were analyzed as censored. Only the first non-persistence was analyzed [18] in the consideration of treatment was interrupted. Figure 1 illustrated the calculation of MPR, PDC, and persistence.

To explore influential factors on statin adherence and persistence, we collected patients’ data from one year prior to the admission for ASCVD and during hospitalization, as well as characteristics of the healthcare providers who prescribed the post-discharge index statin. Patients’ clinical factors included demographics, in-hospital ASCVD diagnosis, intervention procedures received, lipid and electrocardiogram examinations, medications, and history of comorbidity one year prior to the ASCVD admission. Healthcare provider characteristics included the specialty of prescribing physician, the geographic location, and healthcare organization accreditation level. Statins of index prescription were classified according to their capability to lower low-density lipoprotein (LDL): those that could lower LDL by ≥50% were categorized as high-potency (atorvastatin and rosuvastatin), and all other statins as non-high-potency [10]. Dosage prescribed was categorized according to defined daily doses (DDDs) [19] into three groups: ≥2 DDDs as high; ≥0.5 and < 2 DDDs as moderate; and < 0.5 DDDs as low.

### Statistical analysis

Baseline data of patient and healthcare provider characteristics were analyzed descriptively. We presented percentage of good adherence (PDC ≥0.8) and mean MPR every 180 days (equivalent to 6 months) from the index statin prescription to end of follow up. To explore the association of influential factors with adherence while taking into account the inter-relationship among factors and the repeated observations of adherence measurements over multiple 180-day periods of one individual, we used the generalized estimating equation (GEE) to analyze the data [20]. Suboptimal adherence (PDC < 0.8) was analyzed as a binary variable with logistic regression form. Results for the association between factors and suboptimal adherence were presented using adjusted odds ratio (OR) with 95% confidence interval (95% CI). Value of MPR was analyzed as a continuous variable using GEE with linear regression form. MPR mean differences associated with influential factors were presented using adjusted regression coefficients with standard error (SE). The time course of statin persistence was analyzed using the Kaplan-Meier method to account for data censoring, and reported as persistence probability curve. We used Cox’s proportional hazard model to analyze association of influential factors with occurrence of first non-persistence, and reported adjusted hazard ratio (HR) with 95% CI. Two-sided *p*-values smaller than 0.05 were considered statistically significant. Statistical Analysis System version 9.3 (SAS Institute Inc., Cary, NC, USA) was used for the analysis.

## Results

**Fig. 2:**
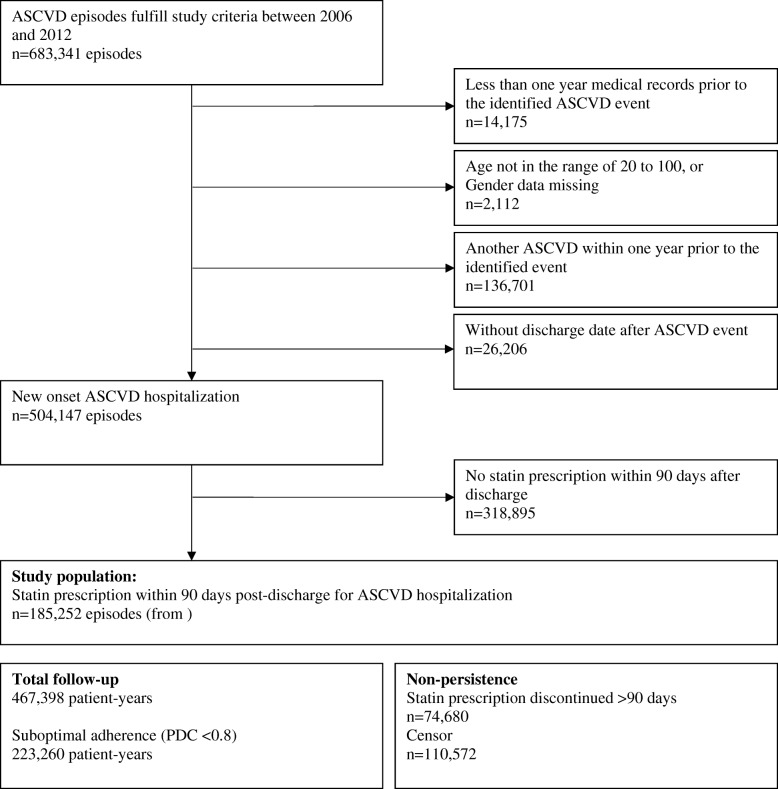
Selection diagram of study cohort

There were 185,252 post-discharge statin initiations identified from 169,624 patients in Taiwan NHI claims database between January 2006 and December 2012. Figure 2 showed the patient selection diagram and formation of study cohort. Their baseline clinical characteristics and healthcare provider characteristics were summarized in Table 1. The mean age of patients at statin initiation was 65.4 years; 66% were males; 82% had received statin during hospitalization, and 47% had received statins during the year prior to the ASCVD hospitalization. Of all the ASCVD hospital discharge diagnoses, there were 74,753 (40%) ischemic stroke, 67,779 (37%) stable CHD with PTCA/CABG revascularization, 33,021 (18%) AMI with revascularization, and 9699 (5%) AMI without revascularization. During the 1-year prior to ASCVD hospitalization, hypertension, ischemic heart disease, hyperlipidemia, and diabetes were among the most prevalent comorbidities; each one of these four were found in more than 40% of the study subjects. Multiple comorbidities were common with a mean Charlson’s comorbidity score of 1.79. Aligned with comorbidity disposition, the five most commonly prescribed drug classes except statin were antiplatelets, calcium channel blockers, β-blockers, angiotensin receptor blockers, and oral antidiabetic drugs.

**Table 1:** Baseline clinical and healthcare characteristics of statin initiator after discharge from hospital for ASCVD

Variable	n	%
Number of events	185,252	100%
Age [mean, SD]	[65.4,12.0]	
Male	122,881	66%
Female	62,371	34%
Statin use during hospitalization	151,020	82%
Statin use one year prior to hospitalization	87,191	47%
Main diagnosis of ASCVD events		
Ischemic stroke	74,753	40%
Stable CHD receiving revascularization	67,779	37%
AMI with PTCA/CABG	33,021	18%
AMI without PTCA/CABG	9699	5%
Examination during hospitalization		
Lipid exam	163,329	88%
EKG exam	146,867	79%
Examinations one year before ASCVD		
Lipid exam		
Number [mean, SD]	[1.59,1.78]	
0 time	65,378	35%
1 time	40,545	22%
≥ 2 times	79,329	43%
EKG exam		
Number [mean, SD]	[1.23,1.72]	
0 time	75,807	41%
1 time	54,008	29%
≥ 2 times	55,437	30%
One year before ASCVD		
Comorbidities		
Hypertension	126,530	68%
Ischemic heart disease	87,031	47%
Hyperlipidemia	81,655	44%
Diabetes	76,390	41%
COPD	18,112	10%
Renal failure	15,988	9%
Liver failure	14,004	8%
Cancer	8877	5%
Depression	8658	5%
Transient ischemic attack	7512	4%
Dementia	4702	3%
Charlson comorbidity score [mean, SD]	[1.79,1.81]	
Medications		
Antiplatelet agents	119,947	65%
CCBs	104,848	57%
β-blockers	92,994	50%
ARBs	72,550	39%
OADs	68,073	37%
Diuretics	56,981	31%
ACEIs	45,236	24%
Insulin	23,958	13%
Anticoagulants	18,080	10%
Digitalis	7514	4%
At index statin prescription		
Healthcare organization accreditation level		
Medical center	95,326	51%
Regional hospital	58,111	31%
District hospital	17,129	9%
Other hospital	10,440	6%
Clinic	4246	2%
Geographic location		
Taipei region	62,979	
Northern region	22,723	12%
Central region	32,740	18%
Southern region	30,274	16%
Kao-Ping region	30,730	17%
Eastern region	5806	3%
Physician Specialty		
Cardiovascular medicine	96,018	52%
Cardiovascular surgery	7540	4%
Neurology	53,050	29%
Metabolism & endocrinology	7899	4%
Internal medicine	13,798	7%
Family medicine	2971	2%
Others	3976	2%

After hospital discharge, most statin treatments were initiated at the outpatient department of medical centers (51%) and regional hospitals (31%), which were the two highest ranks in hospital accreditation level. The treatments were prescribed by physicians with cardiovascular specialties (medicine and surgery; 52 and 4%, respectively), neurologists (29%), and internal medicine physicians (7%).

**Fig. 3:**
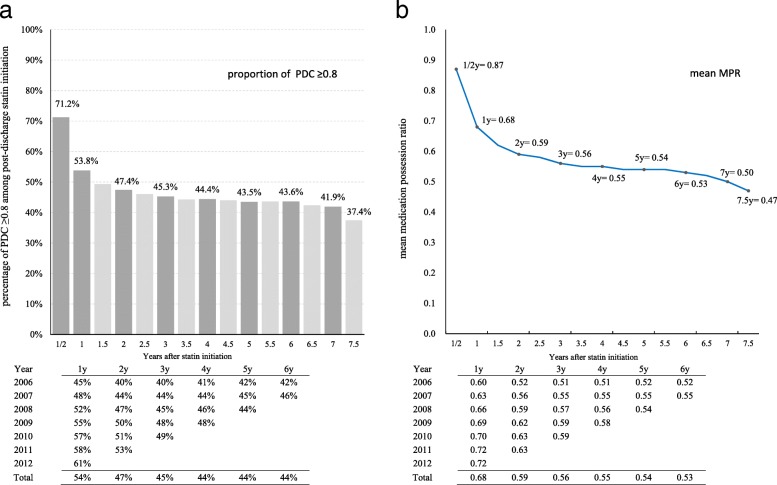
Statin adherence in post-discharge statin initiators in 180-day periods along follow-up; measured by (**a**) proportion of PDC ≥0.8; and (**b**) mean MPR. Two 180-day periods were aggregated as equivalent to one year. MPR: Medication possession ratio; PDC: Proportion of days covered

The total follow up length was 467,398 patient-years. Among these, 223,260 patient-years were classified as having suboptimal adherence. There were 74,680 non-persistence events. Figure 3 showed the trends of good adherence percentage and mean MPR every 6-month from statin initiation to end of follow-up. At the end of the first 6-month period after statin initiation, 71% of patients had good adherence and the mean MPR was 0.87. Good adherence declined to 54% (mean MPR 0.68) at the end of 1 year. Less than half of patients remained on good adherence at end of 2 years with the mean MPR dropping to 0.59. Thereafter between 3 and 6 years, the decline slowed down to about 1% of good adherence or 0.01 for MPR every year. Despite the decline of adherence along longer follow up periods, we found improving trends of adherence over the course of study period. In 2006, 1-year good adherence was 45%, which increased to 61% in 2012; over those same years the mean MPR increased from 0.60 to 0.72. A similar improvement pattern was seen consistently across all follow-up periods.

**Fig. 4:**
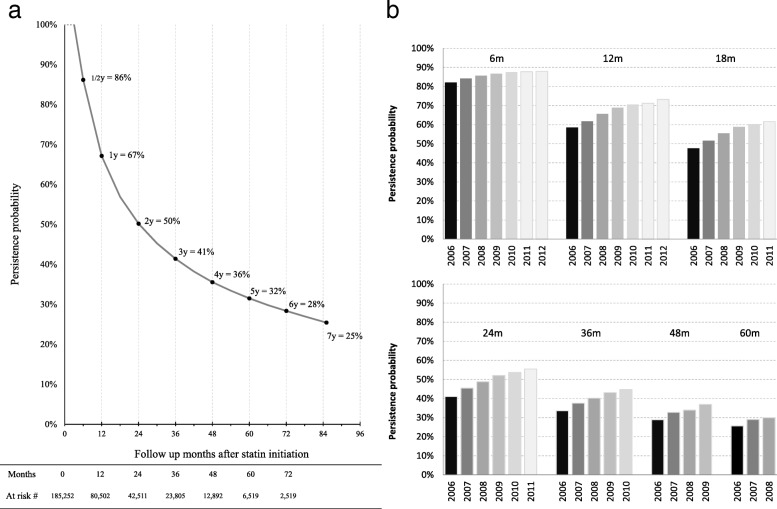
Statin prescription persistence probability in ASCVD events with patients initiated on statin after discharge: (**a**) total patients; (**b**) probability of persistence at 6, 12, 18, 24, 36, 48, and 60 months of follow-up by year of initiation

Figure 4a showed the statin persistence probability curve against follow-up period. Persistence probability was 86% at 6-month following statin initiation. It dropped to 67% at 1 year, continued dropping to 50% at 2 years, and thereafter dropped to 25% at 7 years when the maximum follow-up period ended. The decline was faster at the early period of follow-up: year-over-year persistence probability ratios were 0.75 (for year 2 over 1), 0.82 (year 3 over 2), 0.88 (year 4 over 3), and 0.89 for the remaining years. In spite of the within-cohort decline along follow-up, comparing cohorts by statin initiation year from 2006 to 2012, we observed higher persistence in more recent years: 1-year persistence probability increased from 58% in 2006 to 73% in 2012; at 2-year it increased from 41 to 56%, respectively. Median persistence duration also increased significantly from 16 months to 28 months across the cohorts between 2006 and 2010 (*p* value < 0.05). The pattern of improvement in persistence was similar to that in adherence.

Table 2 showed the associations of factors with potential influence on suboptimal adherence and non-persistence of statin treatment. All the factors were included in the respective models for suboptimal adherence, MPR and non-persistence. Among the healthcare provider factors, statins prescribed in medical center (the highest accreditation rank), by cardiovascular specialists, endocrinologist, and in the more recent years (2012) were associated with significantly less suboptimal adherence or lower non-persistence (all *p* values < 0.01). All the four factors were associated with > 30% lower (adjusted ORs and HRs < 0.7) suboptimal adherence or non-persistence. Among the clinical factors, receiving revascularization procedures in hospital, either for AMI or CHD diagnosis, was associated with less suboptimal adherence (adjusted OR 0.65 and 0.71, respectively, both *p* values < 0.01) and lower non-persistence (adjusted HR 0.69 and 0.71, both *p* values < 0.01). History of previous statin use, either in the year prior to, or during, the ASCVD hospitalization, was also associated with less suboptimal adherence (adjusted OR 0.72 and 0.93, respectively, both *p* values < 0.01) and lower non-persistence (adjusted HR 0.89 and 0.74, both *p* values < 0.01). Patients who received high-potency statins, or moderate dose when compared to low dose, were associated with better adherence and longer persistence (all *p* values < 0.01). On the other hand, clinical factors significantly associated with higher suboptimal adherence or non-persistence were: age > 75 years; female; comorbidities of renal disease, liver disease, depression, chronic obstructive pulmonary disease, or dementia; history in the one year prior to the ASCVD hospitalization of using insulin, digitalis, oral antidiabetic agents, angiotensin-converting enzyme inhibitors (ACEIs), or diuretics (all *p* values < 0.01, expect for female *p* value < 0.05).

**Table 2:** associations of clinical and healthcare provider factors with suboptimal statin adherence and non-persistence in patients post ASCVD discharge

Variable	Adjusted odds ratio	(95% CI)	Adjusted coefficient	(SE)	Adjusted hazard ratio	(95% CI)
Clinical factors						
Patient demographics						
Age at index date > 75 vs. ≤75 (y)	1.10	(1.08–1.12)^‡^	−0.026	(0.002)^‡^	1.11	(1.09–1.13)^‡^
Female vs. male	0.96	(0.95–0.98)^†^	0.007	(0.002)^‡^	1.00	(0.99–1.02)
Previous statin use						
Used one year prior to ASCVD	0.72	(0.71–0.74)^‡^	0.069	(0.002)^‡^	0.89	(0.86–0.93)^‡^
Used during hospitalization	0.93	(0.91–0.95)^‡^	0.015	(0.002)^‡^	0.74	(0.72–0.75)^‡^
Diagnosis of ASCVD events						
Ischemic stroke	Ref.	(0.98–1.07)	Ref.		Ref.	
AMI without PTCA/CABG	1.03	(0.63–0.67)^‡^	0.002	(0.004)	1.06	(1.02–1.11)†
AMI receiving revascularization	0.65	(0.69–0.73)^‡^	0.087	(0.003)^‡^	0.69	(0.67–0.71)‡
CHD receiving revascularization	0.71	(0.98–1.07)	0.075	(0.003)^‡^	0.71	(0.69–0.73)‡
Comorbidities one year before ASCVD						
Renal disease	1.29	(1.25–1.33)^‡^	−0.043	(0.003)^‡^	1.21	(1.17–1.24)^‡^
Liver disease	1.10	(1.07–1.14)^‡^	−0.013	(0.003)^‡^	1.09	(1.06–1.12)^‡^
Depression	1.10	(1.06–1.14)^‡^	−0.015	(0.004)^‡^	1.09	(1.06–1.12)^‡^
COPD	1.07	(1.04–1.10)^‡^	−0.012	(0.003)^†^	1.07	(1.04–1.09)^‡^
Dementia	1.06	(1.01–1.12)	−0.018	(0.005)^†^	1.05	(1.00–1.10)^†^
Transient ischemic attack	0.99	(0.95–1.03)	0.002	(0.004)	1.01	(0.97–1.05)
Ischemic heart disease	0.99	(0.97–1.01)	0.004	(0.002)^†^	0.98	(0.96–1.00)
Cancer	1.00	(0.96–1.03)	0.004	(0.003)^†^	0.98	(0.95–1.01)
Hyperlipidemia	0.93	(0.91–0.95)^‡^	0.022	(0.002)^‡^	0.93	(0.91–0.95)^‡^
Medications one year before ASCVD						
Insulin	1.15	(1.12–1.18)^‡^	−0.020	(0.002)^‡^	1.11	(1.08–1.13)^‡^
Digitalis	1.12	(1.08–1.17)^‡^	−0.022	(0.004)^‡^	1.10	(1.06–1.15)^‡^
OADs	1.05	(1.03–1.07)^‡^	−0.007	(0.002)^‡^	1.09	(1.07–1.11)^‡^
ACEIs	1.05	(1.03–1.07)^‡^	−0.009	(0.002)^‡^	1.05	(1.03–1.07)^‡^
Diuretics	1.05	(1.03–1.07)^‡^	−0.011	(0.002)^‡^	1.05	(1.03–1.07)^‡^
CCBs	1.02	(1.00–1.04)	−0.003	(0.002)	1.04	(1.03–1.06)^‡^
Anticoagulants	0.99	(0.96–1.02)	0.003	(0.003)	0.95	(0.93–0.98)^‡^
β-blockers	0.95	(0.93–0.96)^‡^	0.008	(0.002)^‡^	0.97	(0.95–0.98)^‡^
ARBs	0.95	(0.93–0.97)^‡^	0.008	(0.002)^‡^	0.96	(0.94–0.97)^‡^
Antiplatelet agents	0.94	(0.92–0.96)^‡^	0.010	(0.002)^‡^	0.93	(0.92–0.95)^‡^
Index statin used						
Dosage: moderate vs. low	0.96	(0.94–0.97)^‡^	0.010	(0.002)^‡^	0.95	(0.93–0.96)^‡^
Dosage: high vs. low	1.03	(0.98–1.07)	0.000	(0.004)	0.97	(0.94–1.01)
High potency vs. others	0.91	(0.89–0.93)^‡^	0.020	(0.002)^‡^	0.92	(0.90–0.94)^‡^
Healthcare provider factors						
Accreditation level of statin prescribing healthcare organization						
Primary care clinic	Ref.		Ref.		Ref.	
District hospital	0.88	(0.83–0.93)^‡^	−0.003	(0.006)	0.85	(0.81–0.90)^‡^
Regional hospital	0.78	(0.74–0.83)^‡^	0.020	(0.006)^‡^	0.82	(0.78–0.86)^‡^
Medical center	0.60	(0.56–0.63)^‡^	0.070	(0.006)^‡^	0.61	(0.58–0.64)^‡^
Other hospital	0.76	(0.71–0.82)^‡^	0.033	(0.007)^‡^	0.80	(0.75–0.86)^‡^
Geographic location of statin prescribing healthcare organization						
Eastern region	Ref.		Ref.		Ref.	
Taipei region	0.90	(0.86–0.94)^‡^	0.037	(0.004)^‡^	0.89	(0.86–0.93)^‡^
Northern region	0.87	(0.82–0.91)^‡^	0.030	(0.005)^‡^	0.87	(0.83–0.91)^‡^
Central region	1.00	(0.95–1.05)	−0.001	(0.004)	0.97	(0.92–1.01)
Southern region	0.83	(0.79–0.87)^‡^	0.044	(0.004)^‡^	0.81	(0.77–0.84)^‡^
Kao-Ping region	0.84	(0.80–0.88)^‡^	0.033	(0.004)^‡^	0.84	(0.81–0.88)^‡^
Specialty of statin prescribing physician						
Others (exclude all below)	Ref.		Ref.		Ref.	
Cardiovascular medicine	0.53	(0.50–0.56)^‡^	0.109	(0.006)^‡^	0.62	(0.59–0.65)^‡^
Cardiovascular surgery	0.53	(0.49–0.57)^‡^	0.107	(0.007)^‡^	0.65	(0.61–0.69)^‡^
Metabolism and Endocrinology	0.59	(0.55–0.63)^‡^	0.115	(0.006)^‡^	0.58	(0.55–0.62)^‡^
Neurology	0.68	(0.65–0.72)^‡^	0.065	(0.006)^‡^	0.80	(0.76–0.84)^‡^
Internal medicine	0.77	(0.73–0.82)^‡^	0.040	(0.006)^‡^	0.89	(0.85–0.93)^‡^
Family medicine	0.84	(0.77–0.92)^‡^	0.020	(0.008)	0.92	(0.86–0.99)^†^
Year of index statin prescription						
2006	Ref.		Ref.		Ref.	
2007	0.89	(0.86–0.91)^‡^	0.024	(0.003)^‡^	0.91	(0.89–0.94)^‡^
2008	0.83	(0.81–0.85)^‡^	0.036	(0.003)^‡^	0.86	(0.84–0.88)^‡^
2009	0.76	(0.74–0.78)^‡^	0.048	(0.003)^‡^	0.80	(0.78–0.82)^‡^
2010	0.72	(0.70–0.74)^‡^	0.055	(0.003)^‡^	0.75	(0.73–0.77)^‡^
2011	0.69	(0.67–0.71)^‡^	0.059	(0.003)^‡^	0.73	(0.71–0.75)^‡^
2012	0.49	(0.47–0.51)^‡^	0.100	(0.003)^‡^	0.69	(0.66–0.72)^‡^

†*p*-value < 0.05;^‡^*p*-value < 0.001. All factors were included for adjustment

## Discussion

In the present study, we found early and steep decrease in treatment adherence and persistence soon after statin initiation. By the end of the first 6 months, 14% of patients initiated on statins became non-persistent. According to the non-persistence definition of reaching 90 days without statin prescription covered, these patients would not have used statin for over three months. Moreover, 1 in 3 patients discontinued statin prescription after 1 year. These findings should raise concern for the ASCVD patients, who had very high risk of recurrent cardiovascular disease and related mortality. This early discontinuation of therapy has also been widely observed [6, 21–25]. The reported drop in adherence and persistence ranged from 60% over 12 months in primary care setting to 16% at 1 month after non-ST elevation acute coronary syndrome. The issue of treatment non-persistence was more serious in real-world practice than was reported in clinical trials. Benner et al. [6] reported that the 5-year discontinuation rates ranged from 6 to 30% in clinical trials, whereas the discontinuation at 5 years in the present study was 68%, which was at 2 to 10 times higher than the rates from clinical trials. Alongside high non-persistence, adherence to treatment was suboptimal. For example, only 45% of patients retained good adherence at 3 years. This result was consistent with the earlier Taiwanese study by Li et al. [5], who found a 41% statin adherence (measured as MPR ≥80%) at 3-year follow-up in patients with first statin prescription from year 2001 to 2007.

Despite the early decline and suboptimal statin treatment adherence and persistence, we observed improving trends in recent years across our study period. There was a 16% improvement in good adherence at 1-year among those who initiated statin in 2012 compared to those initiated in 2006 (61% vs. 45%). This trends were consistent across all measurements, including: a higher 1-year mean MPR (0.72 vs. 0.60), improved 1-year persistence (73% vs. 58%), and longer median duration of prescription persistence (28 months vs. 16 months), in the respective years. These improvements over study years were independent of other factors included in the multiple variable analyses. The increased use of statin in more recent years has been reported in studies from several countries, including UK [26], Canada [27], Germany [23], and US [2, 6] in the 2000s and earlier. Authors attributed the increased use of statin to the publications of pivotal statin trails during these periods. More evidence from studies that were published thereafter supported the use of more intensive statins, especially in the secondary prevention of cardiovascular disease, which might have influence on the trend of higher adherence and longer persistence of statin prescription.

In a statin utilization study, Jackevicius et al. found cardiologists, who presumably were more up-to-date on new information in the relevant field, increased satin prescriptions more than non-cardiologists did after the publication of the Scandinavian Simvastatin Survival Study [27]. Consistent with this finding, we found the statin prescribed by cardiologists were more likely to have good adherence and longer persistence, which was independent of patients’ diagnosis, intervention procedure received and co-morbidity in multivariate adjusted analyses. The specialty of prescribing physician was among the most influential of analyzed factors on statin adherence and persistence. Statin treatments prescribed by cardiologists who also worked in medical centers, the highest accredited healthcare organizations in Taiwan, were 3 times more likely to have good adherence: OR = (0.53 × 0.60)^− 1^, or had 2.6 times fewer chances of non-persistence: HR = (0.62 × 0.61) ^-1^ than those prescribed by physicians of other specialty in primary care clinics. The influence of healthcare providers on adherence to treatment has also been reported in previous studies. In a review article on the importance of medication adherence on cardiovascular outcome, Ho et al. pointed out there were the highest risk of non-adherence when patients in the transition period from hospital discharge to the outpatient setting [16].

However, a recent study in Norway found no overall change in adherence when primary healthcare physicians took over the prescription responsibility at approximately three months after AMI [28]. One possible explanation might be related to the dissemination of new information, as there could be a lag for the information to reach non-cardiology physicians and the broader healthcare institutions beyond top-ranked medical centers. After a couple of years of information propagation, the knowledge dissemination could be saturated across the healthcare provider community. This could partially explain the slowdown on improvement of 6-month statin persistence, which reached a plateau after the year 2010 at the level of 88% in the present study. This hypothesis might also explain the plateau of adherence to secondary preventive medications prescribed by primary healthcare physicians observed in the 2016 Norway study [28]. Consistently, one recent New Zealand national data linkage study of statin maintenance in patients after acute coronary syndrome found better and stable adherence: the percentage of patients with MPR ≥ 80% was 69% in first year and 66% in third year [29]. Statin treatment adherence was in general improving globally. This was in alignment with the consistent recommendations of using statin by many international treatment guidelines across the world [9–11, 30]. These guidelines play an important role in educating prescribing physicians. However, the remaining 12% short term discontinuation in the present study might be due to reasons other than knowledge dissemination, and more studies would be warranted to identify potential intervention strategies for further improvement.

In addition to the healthcare factors (physician specialty and healthcare organization accreditation level), we also included other factors that might be associated with statin adherence and persistence in the present study. These factors can be organized according to the framework proposed by the World Health Organization (WHO), which classifies factors related to chronic disease treatment adherence into five dimensions [3]. We mapped the variables captured in our database to the five dimensions: age and sex for “patient factors”; diagnosis of index ASCVD event, comorbidities, and use of other medications for “condition factors”; prior use of statin, history of lipid examination for “therapy factors”; geographic location and year of index prescription for “social/economic factors”; and physician specialty and healthcare organization accreditation level as mentioned above for “healthcare system factors”. According to a systematic review of 28 statin adherence studies conducted by De Vera et al. [31], most studies were not able to include factors for all five dimensions. This was one of the strengths of the present study, as a wide range of influential factors on treatment adherence and persistence were captured in the database we used in our analysis.

There were several other strengths with our study. The NHI database is an ideal source of data for drug utilization research. Because of the nationwide, universal coverage, and completeness of population prescription records, we minimized the risk of selection bias due to insurance eligibility, variations in benefit provided by different health plans, or incomplete follow-up due to a patient changing from one healthcare plan to another. The universal coverage provided a link between hospitalization data and post discharge outpatient prescriptions, which gave a complete picture of a study cohort that was more relevant to patient care. Because of the huge size of the database, in which there were sufficient number of subjects with target ASCVD events, and the complete prescription records tracking all visits for each individual, the analysis results were highly reliable. The database captured the outpatient prescriptions on a daily basis, and we retained the time sequence information to analyze the time course of adherence and persistence status. This is different from the previous study by Li et al. [5], who pooled all the prescriptions across the entire study period into one aggregated measure for adherence, and was not able to show the time pattern of adherence. Also owning to the large number of prescriptions tracked, we were able to group prescriptions into series of equal length 180-day follow up period that minimized the potential variation in the calculation of PDC and MPR due to different cohort time spans [32]. And lastly, in addition to the use of both MPR and PDC, which are the two common measurements for adherence, we analyzed statin persistence using time to first non-persistence, providing a finer picture of treatment continuity. Sustained treatment continuity is very important in preventing recurrent cardiovascular attack, and as such is highly sensitive to short term interruption of statin treatment. Our study showed that only 1 in 4 patients maintained long-term treatment persistence at end of the 7-year follow-up, which was much lower than the 42% patients with good adherence or 0.5 mean MPR at the same time point. Persistence as an indicator revealed greater urgent needs to maintain stable treatment for ASCVD patients.

There were limitations with this study as well. Firstly, we used the prescription records instead of other direct measurements such as level of statin or its metabolites in blood or urine. A direct measurement is indeed more relevant to treatment effect. However, the measurement of stain or its metabolites in patients’ blood samples were not a routine practice. The cost and difficulty to conduct direct measurements in a population of 23 million people for 7 years of study time span with sufficient frequency would be extremely huge. In contrast, analyzing the available prescription records, we took the advantage that all the statins were reimbursed and all the statin prescriptions should have been captured during the study period for the whole population. If there was no prescription record for a patient, there was very little chance the patient would have taken the medication. Thus, the observation in this study could be considered as optimistic in treatment adherence. Bearing this in mind, the actual treatment adherence might be even worse and should be taken more seriously. Secondly, we did not have access to laboratory test results such as serum lipid level, as the reports were not required for reimbursement and not captured in the database. Serum lipid level was commonly measured to guide statin prescription. One particular concern was that the old reimbursement guidelines required physicians to reduce the dosage of statin once the lipid level reached the treatment target. This might be one reason for the higher suboptimal adherence and non-persistence on statin treatment at primary care clinic and in the earlier years. The reimbursement guidelines have been revised to remove the dose reduction requirement in 2013, aligning with international treatment guidelines’ recommendations to maintain high-intensity statins for secondary prevention of cardiovascular disease [9–11]. Finally, there were no reliable data regarding patients’ intolerance to treatment, which limited our ability to evaluate the impact of this patient-specific factor on treatment adherence and persistence.

The evidence on efficacy of statin treatment for secondary prevention of cardiovascular disease was solid and used by most recent international treatment guidelines [9–11] and Taiwan guideline [30] for the recommendations of statin use. However, medications could not be effective to treat patient if they were not taken. The issue of treatment adherence and persistence should be addressed by healthcare providers and policy makers in order to develop and implement effective intervention to optimize patient care. The data on adherence and persistence in Asia were relatively scarce compared to those in western countries. Our study provided a nationally representative and comprehensive picture on statin treatment adherence and persistence in ASCVD patients, quantified their associations with factors across multiple dimensions, and identified opportunities for improvement. Further research is warranted to validate the effectiveness of patient care quality improvement measures and to continuously monitor patient outcome.

## Conclusions

Despite the improving trends of statin treatment adherence and persistence in the recent years, they were still suboptimal in Taiwan. Only 1 in 4 of these high risk patients who initiated statin treatment maintained continuous persistence on treatment at seven years, which should raise our serious concern. Among multiple dimensions of factors related to treatment adherence and persistence, association was the strongest with healthcare provider factors. This study provides the evidentiary basis for further investigation on the effectiveness of interventions to improve statin adherence and persistence and to develop more effective patient management strategies.

## Abbreviations

95% CI: 95% confidence interval
ACEIs: Angiotensin-converting enzyme inhibitors
AMI: Acute myocardial infarction
ARBs: Angiotensin receptor blockers
ASCVD: Atherosclerotic cardiovascular disease
CABG: Coronary artery bypass graft
CCBs: Calcium channel blockers
CHD: Coronary heart disease
COPD: Chronic obstructive pulmonary disease
DDD: Defined daily dose
EKG: Electrocardiography
GEE: Generalized estimating equation
HR: Hazard ratio
ICD-9-CM: International Classification of Diseases, 9th Revision, Clinical Modification
LDL: Low-density lipoprotein
MPR: Medication possession ratio
NHI: National Health Insurance
OADs: Oral antidiabetic drugs
OR: Odds ratio
PDC: Proportion of days covered
PTCA: Percutaneous coronary intervention
SAS: Statistical Analysis System
SD: Standard deviation
SE: Standard error
WHO: World Health Organization
